# A Dissertation on Local Affections of the Nerves, Drawn up under the Inspection of M. Beclard; with Communications from Dupuytren, Marjolin, Richerand, Roux, &c.

**Published:** 1827-04

**Authors:** 


### VIII.

*Dissertation sur les Affections Locales des Nerfs, par* Pierre Jules Descot, *d'un Travail fait sur la Direction de M. Béclard, et enrichi de nombreuses Observations, fournies par M. M. Béclard, Dupuytren, Marjolin, Richerand, Roux, Moreau, Ribes, Richard, Bérard, Bogros, and Briquet.* Octavo, pp. xx. 336. A Paris, 1825.

Physicians see few diseases, indeed, in which the nervous system, including the brain, spinal marrow, the nerves and their ganglions, is not implicated in one degree or other: next to the sanguineous,* on which it is dependent for its existence and vitality, it requires much consideration in treating almost every affection of the animal economy. Important, however, as the subject is, we intend not at present to enlarge our article by introducing a sketch of the facts and philosophy on which this comprehensive statement is founded; or, as might be expected, by entering into the details of a neuropathic bibliography: such topics would undoubtedly lead to very interesting discussions; but we have chosen, for this time at least, to confine ourselves to a purely analytical exposition of that branch of the nervous pathology which it is the chief object of Dr. Descot's dissertation to explain. At once, therefore, we enter on our task, without farther remarks, either apologetic or preliminary.

Additionally to the usual pageantry of a book, a dedication, an "avertissement," and a preface, Dr. D. has premised a discourse intended to have been spoken by him over the remains of his patron and friend, Professor Béclard, on the day of that illustrious man's funeral. There can be no doubt of our author's having regarded his "Discours" as a master-piece of pathetic elocution: nevertheless, we acknowledge that it is not much to our taste, and we felt unable even to suppose what could have been the writer's inducement to give it a place in the same volume with this simple inscription—"Professori Desiderato, Béclard, silentium verbis fecundius, P. J. Descot." With this hint in passing, we advance to the Dissertation itself; and, from the xv. chapters it comprehends, shall proceed to draw such facts and doctrines as may appear best adapted to promote the extension of medical knowledge and experience.

Chap. I.—*Of the Nerves.* This, of course, is entirely anatomical,

---

* Blood-vessels require an uninterrupted supply of the "nervous energy" for maintaining perfectly the discharge of their incessant functions; but there is no evidence in physiology from which we can *assert* that the nervous energy is in any form or degree imparted to the blood itself: this last is the communicative source, from being to being, of substance, vitality, and mind.

and describes the organization of the nerves themselves, their ganglions, anastomosings, blood-vessels, neurilematous envelope, functions, and distribution. We do not deem it necessary to dwell on this section; but we must advert to this statement of Dr. Descot's, inasmuch as we regard it as being rather remarkable, "Nerves," he says, p. 11, "at their insertion into the cranial and spinal brains, are somewhat thickened and very soft; at their other extremity, *their size is much more considerable still and their softness very great:*" if there is no error of expression or description here, we would ask,—are the facts demonstrable to the observation of ordinary enquirers?

According to our author, each nervous fibre is formed of a white, pulpy substance, consisting, like the nervous structure in general, of an assemblage of microscopic globules, and a neurilematous envelope, which supports the medullary substance, and never "abandons" it but at the two extremities and in the ganglions. Blood-vessels, very large, relatively to the nervous fibres, are distributed to their envelope, and penetrate into their internal structure. Nerves possess an extreme tenacity, depending on their neurilema, which renders them exceedingly difficult to be ruptured. The medullary matter, of which they essentially consist, is immoveable in their centre, and we cannot suppose that it there experiences "*un mouvement de translation;*" partly on account of its consistence, or at least its great viscidity; and partly because the neurilema is not a real canal, since the cellular texture which forms it dips into the pulpy substance of the nervous fibre. We resign the interpretation of these aphorisms to those of our readers who have experience in practical anatomy.

In a foot-note to p. 12, Dr. D. makes the announcement, that M. Bognor has recently submitted to the Academy of Sciences, a memoir on the structure of the nerves, in which he states, as the result of his experiments and researches,—*that the nerves have a central canal:* we agree with him in adding, that if this fact shall be well established, it will necessarily exert great influence on the physiology of the nervous system. He then goes, at some length, into a statement of the experiments made by Dr. Majendie on the roots of the spinal nerves, and this of course leads him to notice the claims of Mr. Charles Bell to the priority of discovery. Dr. M. he says, had just finished and published his experiments when he understood that Mr. Bell had, thirteen years before, divided the roots of the spinal nerves and discovered that this section did not prevent *motion;* and that this last anatomist had consigned this result to a printed, but unpublished tract, which was distributed among his friends. From a perusal of this tract, Dr. D. concludes that Mr. Bell, conducted by his ingenious ideas on the nervous system, had well nigh discovered the functions of the spinal roots: nevertheless, the fact, that the *anterior* are destined to *motion*, whilst the *posterior* belong particularly to *sensation*, appears to have escaped him: it is to the having established this fact, in a positive manner, that M. Majendie limits his pretensions. Dr. D. repeated these experiments on young dogs, and found them invariably to afford the same results.

The nerves, he also informs us, which traverse ganglions and those that terminate in viscera and the sides of vessels, do not transmit impressions, and the will does not direct the motions which they determine: it is only in strong affections of the mind and of these organs, that their functions produce sensations, and the motions occasioned by nerves are disturbed. It is, he thinks, to the inter-communications of nerves that sympathy ought in a great measure to be referred.

Dr. D. concludes his first chapter with stating that the diseases of the nervous system may be divided into those which, by affecting the brain and spinal marrow, introduce disorder, more or less extensive, into the functions of the whole frame, and into those which, by implicating a single nerve, do not necessarily disturb the rest of the system: he has selected the last class as the subject of his dissertation, to the next section of which we now accompany him.

CHAP. II.—*Of Wounds of the Nerves.* Wounds of nerves, Dr. D. says, are confounded with all lesions of this kind, and occasion the concomitant pain; but, he adds, we should regard those only as wounds of nerves, which affect a branch or appreciable fibre, and not the slender extremities of these organs. Such injuries, whatever be their seat or degree, are immediately followed by intense pain, whether the nerve be perfectly or partially divided: they are not necessarily accompanied by palsy of the parts to which the nerve is distributed; but when palsy does supervene, it may be temporary or permanent. According to circumstances, the wounded parts sustain *adhesive* inflammation, ending in "primitive re-union:" occasionally the *suppurative* succeeds, and sometimes the *ulcerative*, which, becoming chronic, leads to various alterations of texture: not seldom, these diversities of inflammation appear to depend on the kind of traumatic lesion; and often on accidental causes, as motion of the injured part, and the influences of the atmosphere. The constitution of the patient, and his previous state of health or disease, always modify the effects of wounds of nerves as much, or even more, than those of any other lesion. These remarks lead him to examine successively the different kinds of the wounds of nerves, and to—

CHAP. III.—*Puncture of Nerves.* On the instant of a nerve's being punctured or partially divided, there is always a very acute pain in the parts to which the nerve and its branches are distributed; but the consequences of the wound are exceedingly variable. Most frequently, when the person is healthy, keeps himself tranquil, and avoids every other cause of disease, the wound heals readily and without accident. Dr. D. confirms this statement by two experiments, which, as they are concise, we shall transcribe.

*Exp. 1st.* Dr. Descot punctured the sciatic nerve of an active young pointer dog, after having exposed and separated it from the surrounding parts: the edges of the wound were kept in apposition by a single suture. When the puncture was inflicted, the animal exhibited symptoms of enduring exquisite pain; but, during the day, he lay in a squatted position, and seemed quite easy: next day he was incapable of supporting himself on the wounded limb: on the third, fourth, and fifth, he kept the lying posture, but was easy, and took food: on the sixth, he began to lean on the paw of the lesioned member, which only bent under him in walking: on the ninth, he had recovered his former vivacity, and the perfect use of his leg; was very lively, ran and leaped: on the 13th, his appetite, spirits, and general condition were excellent: and, by the eighteenth, no trace of the operation remained.

*Exp.* 2*d.* Another dog had the left sciatic nerve subjected to the same treatment, which produced similar results: by the twelfth day, he had perfectly recovered the power of walking, without either lameness or the least stiffness of the member: on the twenty-fifth day after the operation the parts were examined, when the nerve was found to be in its natural condition; only, about three or four lines above its division into the internal and external popliteal branches, there was a point somewhat thicker and more opaque than the rest of the nerve: this slight enlargement occupied one of its sides and a part of its anterior face.

Serious accidents, our author goes on to state, when they do supervene, depend on the wound only as an occasional cause: in short, he says, puncture of a nerve is sometimes accompanied or followed by very intense pain in the wounded place; sometimes even by convulsions, which, as well as the pain, extend to distant parts, and occasionally implicate the whole system. These positions are illustrated by two histories of punctured nerves, in opening the external jugular vein, by Monfalcon; one from bleeding in the foot, and another from a wound of the saphenal vein and nerve, by Sabatier; one, from Mr. Swan's work, of a cutaneous nerve of the arm injured in venesection; and one by Dr. Verpinet, by a condensed version of which we shall finish our account of this section.

*Case.* A young lady was accidentally wounded in the inferior and external part of the fore-arm about two inches above the thumb: the puncture measured about four lines in depth. The surgeon, having stopped a slight hæmorrhage, and believing that the acute pain which was felt might soon subside, endeavoured to heal the wound by the first intention: nevertheless, the pain continued with unabated intensity, not only in the fore-arm, but also in the thumb and extremities of the fingers. Sometimes it yielded slightly to the use of volatile and anodyne liniments, but it soon revived. The arm, at last, became the seat of lancinating pains and spasmodic movements, and even of convulsions: voluntary motion in the thumb and fingers became imperfect, occasionally impossible: changes of the atmosphere exerted a remarkable influence on these symptoms; they decreased in dry weather, and were aggravated when it was cold and moist: the north and north-westerly winds likewise caused injurious effects. Some benefit was derived from the baths of Bourbonne, and from pumping its waters on the arm; but, in a short time, the symptoms re-appeared with extreme violence, the spasms became general, and the patient began rapidly to sink. M. Petit, of Lyons, was consulted: he advised the application of a "compressive" bandage and fomentations to the cicatrix, with an opiate mixture; and, if these means should prove unavailing, to destroy the parts originally lesioned with the actual cautery. The first set of remedies was employed for a month, without the least advantage; the other, on the contrary, determined results as fortunate as they were unexpected. Three applications of the cautery produced an eschar, which was soon detached; simple dressings succeeded; and the cure of a disease which had rendered exquisitely miserable the existence of a young person, alike remarkable for meekness and fortitude, was, from that time, complete.

CHAP. IV.—*Division of Nerves.* Section of a nerve, by a cutting instrument, is accompanied with severe pain, and instantly followed by insensibility of the skin, or paralysis of the muscles to which the nerve is distributed: its two ends, at the same time, are a little separated, and the space between them is increased by some motions and diminished by others. In some cases of this kind, says Dr. D. on the margins of the wound being placed in apposition, and the parts kept at rest, reunion has been immediate and perfect: in others, though less frequently, very serious consequences have supervened. We transcribe the author's practical demonstration of this doctrine.

*Exp. May 22d,* 1822. Having exposed the right sciatic nerve of a little spaniel, he divided it in two places, about half an inch distant, and allowed the intermediate portion to remain: the wound was re-united by the first intention. The animal appeared to suffer great pain, followed by howlings, but with little agitation: immediately after the operation, it had general shivering; in the evening, shivering, dullness of the eyes, depression, and defective appetite: it could not lean on the wounded limb, which it trailed in walking, but, when standing still, kept bent and carried forward: having taken some drink it lay down, apparently much exhausted. On the 25th, the exhaustion and thirst continued; appetite slightly improved; the limb in the same state: next day there was great indifference to food: on the 28th, it began to lean upon the limb in walking, and voided its urine: on the subsequent day, it was overturned by another animal, and uttered plaintive cries; on the next, it walked with greater ease and had better appetite: and, by the 31st, it continued to use the limb, which, however, made a short step in walking.

*June 1st.* The left sciatic nerve of the same animal was simply divided by a transverse incision, which occasioned acute pain; in the evening, spasmodic motions were observed in the left fore-paw; next day, the action of both the hind legs seemed to be alike in walking, notwithstanding the operation formerly performed on the right leg; the animal was moderately tranquil, and endeavoured to scratch itself with the left hind foot. This state continued till the sixth, when it had an improved appetite, and walked with the claws of its wounded limb bent under the paw: on the eighth, it was dull, and appeared to be unwell; it trailed the hind legs in walking, and had no appetite; on the tenth it would not rise, and refused all kinds of food. These symptoms increased, with emaciation, debility, difficult respiration, and "engorgement" of the nostrils, till the twenty-second, on the night of which the animal died.

*Examination of the right Sciatic Nerve.* The two incisions were re-united by cicatrices, somewhat smaller than the rest of the nerve, which was whiter, firmer, and thicker, above than below the higher section: above this was a small gangliform enlargement, without any thing of the kind above the lower: the tissue of the cicatrices had a rose colour, but the usual nervous fibres could not be detected in it: the texture of the nerve itself was red and very vascular, and its fibres much less distinct below the two incisions than above the "first."

*Left Sciatic Nerve.* At the place of its division, the nerve was re-united by a cicatrix, more than three lines long, and evidently smaller than the rest of the trunk: above the section, it was white and compact, and slightly thickened; below softer and redder: examined with a magnifying glass, the inside of the cicatrix had a very red colour; below this, less so; above it, red only at the enlargement. *N. B.* Dr. D. has preserved these portions of nerves, and also the animals on which the experiments were made.

*Abdominal Organs.* The mucous membrane of the stomach and intestines was paler than is natural; the stomach contained a large quantity of frothy matter, but no traces of food; in the duodenum was found a little "clear"-coloured bile; the bowels were empty; the liver reddish-brown, crepitant, and easily torn.

Next in order follows a case by Professor Beclard, in which the cubital nerve, artery, and adjacent tendon, were divided by a sharp instrument: the wound was dressed and healed by the first intention, with recovery of sensation and all the functions of the parts. Our author has known such treatment of a divided nerve in the forefinger followed by equally happy effects; and so have we, and so has every body. It would be important to know the exact state of the nerves in regenerated parts and members restored by the processes of adhesion.

In traumatic inflammation, the two ends of the nerve, especially the superior, swell and become vascular; the surrounding cellular texture also inflames and becomes compact: the swelling and redness of the nerve extend in the end above the wound, but not in that below it: the small space between them is filled with the investing cellular texture, which contracts intimate adhesions with them: ultimately, the inflammatory redness dissipates; the enlargement of the nerve remains; and the cicatrix unites the two thickened extremities. When there is much loss of substance, the ends of the divided nerve continue separated in proportion to the absence of structure: these and the intermediate cellular texture experience each of the changes already described. When the loss of substance is inconsiderable, the two extremities come to be united by a cicatrix less thick than themselves: its thickness, in general, is proportionate to the defect of substance: when this is considerable, the separated ends of the nerve grow rounded, the upper presenting a large, the lower a small enlargement, and both dip into the common cellular texture. Dr. Descot adds, in a note, that when simple section of the other nerves, such as the cubital and the pneumo-gastric, is practised, the separation is usually not so great as in division of the sciatic: the cicatrix is then thicker *(moins etroite)* and the two ends of the nerve are more intimately blended. The results of another vivisection, and the dissection of a tetanic patient, are detailed in confirmation of these doctrines, but, as much important matter is still before us, we must pass these with a cursory reference.

Simple section of a nerve, in a part subjected to much motion, or where motion occasions very considerable separation of the nerve's divided ends, is followed by the same effects as section with loss of substance. When two separate sections are made, two corresponding cicatrices result; but the intervening portion of nerve shrinks and becomes smaller. After amputation, an "ovoid tubercle" is formed on the extremity of each divided nerve: this tubercle is usually three or four times thicker, and much firmer, than the nerve itself; has a very different texture, but the same colour: its tissue is entirely fibro-cellular, in which the nervous filaments grow imperceptibly finer and disappear, so as to be incapable of being distinctly traced to its extremity. Soemmerring refers to such tumours, the pains experienced in the cicatrices of amputated members, at the time of atmospherical changes: in his opinion, they swell in consequence of "hygrometric absorption," and thus exercise pressure on the medullary substance of the nerve.

CHAP. V. "*Distension*" *and Laceration of Nerves.* Violent distension of a nerve, by means of motion, is rare; its rupture impossible, without an accompanying over-stretching or laceration of the parts in which it is situated. In some instances, as in cases of chronic exophthalmy and palsy of the deltoid muscle, nerves may be slowly and progressively elongated, without their tissues being affected or their functions much deranged: sudden distension, on the contrary, often produces most important injury—over-extension, laceration, and a train of formidable symptoms. Dr. Descot instituted various experiments on animals, from the results of which he infers that distension or the violent rupture of a very large nerve, does not occasion serious effects, unless when the lesion has place "far up" in the thigh, or in the pelvis: the history detailed by him is instructive, but too long for our limits.—Fontana and Orfila have shewn, that the poison of the viper does not determine its characteristic effects when applied exclusively to the nerves.

Chap. VI. "*Commotion*," *Contusion, and Contused Wounds of Nerves.* When bones sustain a concussion, the nerves which pass along them are implicated, and become the seat of a kind of stupor or numbness, with a greater or less degree of pain. The general "commotion," our author says, experienced by a member when struck by a bullet or other projectile moving with great force, depends solely, or at least principally, on the "commotion" of its nerves. Contusion, whether effected by compression or percussion, "always" determines solution of continuity in the more delicate parts of organs, whilst the others escape: such lesions give rise to the extravasation of fluids which are infiltrated or diffused into the parts that continue unimpaired; and these two causes of irritation—the "contusing" action itself, and the effusion resulting from it—excite an inflammation in the injured parts, which terminates in resorption of the extravasated fluids, and reunion of the divided textures: sometimes, however, it gives place to suppuration, or subsides imperfectly, and thus continues a "centre of pains and other accidents." What remains of this section is occupied with the outlines of fifteen pathological histories, including an experiment of our author's on a living dog, and a sketch of the subsequent dissection: most of them are selected from the books of eminent writers; we abbreviate one communicated by M. Ribes, as containing some facts very worthy of attention.

*Case.* Lesueur, aged 45, was wounded with a musket-ball, in the calf of his left leg, at the battle of Wagram, and lay till the following day on the field: much swelling of the parts ensued and prevented extraction of the bullet, which was evolved at the end of three months, and the wound soon afterwards cicatrized. On the 18th day after the accident, he began to exhibit a train of very extraordinary nervous symptoms, which resisted a great diversity of means for more than eight years. It was observed that, some days before each accession, a tumour about the size of a pullet's egg arose over the cicatrix, and felt painful on pressure: the leg became livid, and the patient's gait unsteady: by and by, the wounded member could not sustain his weight: convulsions supervened; and, beginning at the cicatrix, they affected all the left side of the body, ultimately implicating the right: the lower extremities were alternately bent at the knees, and extended; and these motions ended in a transient tonic contraction. In a short time, the arms ("*membres thoraciques*") underwent the same contractions; and then, says the reporter, the unhappy man became a prey to the most excruciating distress; he felt as if his bones were bruised, and the "nervous cords" experiencing laceration. He declared that his limbs were burning, and expressed his agonies by loud and fearful cries; on these occasions, a profuse sweat issued from all the pores of his body, and moistened the bed-clothes. His lower jaw was affected with tremulous motions, like those which have place in the cold fit of an intermittent: the abdominal muscles were not particularly contracted, but the posterior cervical sometimes sustained a certain degree of rigidity; the pulse was frequent and concentrated; respiration preternaturally rapid; the intellectual faculties unimpaired; and he was tormented with unappeasable thirst. The subsiding of his paroxysms was announced by quick extensions and flexures of his limbs, which continued quiet in the last state for a few seconds, and then underwent the same contractions: in the end, a calm succeeded, but the patient required to remain some hours in bed to recruit his exhausted strength.

Lesueur was admitted into the "Hôtel des Invalids" on the 4th Nov. 1810; and, during the first six months, had daily accessions of his disease; their invasion, intensity, and duration, were uncertain; the longest paroxysms never exceeded three hours; after this period, he had a calm of forty days, in which he seemed quite delivered from all his distresses; but a new seizure manifested itself in daily attacks, and confined him to his bed for four months. At last, from August, 1812, to October, 1817, the intermissions were longer, being generally from ten to twelve weeks; but to this state of tranquillity succeeded an accession which lasted forty days, accompanied with occasional seizures, excited and aggravated by the least error of discipline, moral as well as physical. In the intervals between his paroxysms, this man never enjoyed a perfect calm; he felt himself in a condition he could not define; shudderings and startings interrupted his sleep; he had frequent palpitations and sweats, which, though less considerable than during his seizures, were such as gave him much inconvenience; he was restless, irascible, hard to please, always wanting something, and never content.

Among the multitude of remedies exhibited for this man's cure, opium and moxa only were productive of any advantage; bathing proved utterly useless. At last, in a consultation, about the middle of October, 1817, it was resolved to attempt relieving his sufferings by an excision of a "very large piece" (nearly two inches) of the external popliteal nerve. This was done, with much dexterity, by M. Yvan, surgeon to the hospital; and, by the eighth day afterwards, the wound had cicatrised. Having slept a few hours after the operation, he declared that his condition was altogether changed; that he experienced such a total revolution, as to be no longer the same person; he was composed and tranquil; only he felt a pain in his foot which was entirely new, but this soon subsided; the sweatings, palpitations, startings, and shudderings, which formerly disturbed his sleep, also disappeared; sensation and motion, however, were lost to the parts over which the nerve is distributed. Since the operation, eight years, he has had six or seven accessions of the neuralgy, but in a much milder form; they were induced by fits of peevishness and anger: his health was otherwise good.

CHAP. VII.—*Wounds of Nerves, with Lodgment of Foreign Bodies in them.* Nerves are so small in size, says Dr. D. that we rarely find foreign bodies, introduced by means of a wound, remaining in their structure: he gives three cases from the Medico-Chirurgical Transactions, the Medical and Physical Journal, and the private communications of Professor Béclard; in which accidents of this kind were followed by exquisite symptoms: of this last, we subjoin a condensed translation.

*Case.* A man, about fifty years of age, had a large aneurism in the ham, for which the femoral artery was tied near the middle of the thigh: a flat ligature was applied, cut short, left 60 hours in the wound, and then withdrawn: pulsation instantly subsided in the tumour, and never returned; but the blood's circulation was not re-established in the lower parts of the member, and gangrene supervened: it seized the foot, and greater portion of the leg. "Separative inflammation" now exhausted the patient, and brought his life into imminent danger: this led to amputation of the thigh; and, among the "multitude" of superficial arteries requiring to be tied, was that which passes between the branches of the ischiatic nerve. The wound healed almost entirely by the first intention, and the cure was nearly complete when tetanus assailed the sufferer and destroyed him, notwithstanding the employment of many energetic remedies. On inspection, the arteries generally were discovered to be indurated and ("*calcaires*") ossified: the sciatic nerve, on being examined at the stump, exhibited a considerable enlargement, which included the knot of a ligature that had not been detached. This person had formerly sustained an injury of the radial nerve. Does the author, by making this remark, propose to intimate that frequent nervous lesions tend to induce the tetanic predisposition?

Dr. Descot is of opinion that the effects produced on the nerves by affections of the teeth may have some relation to those determined by the lodgment of foreign substances; and, out of a great number of facts confirmative of this proposition, relates one communicated by M. Briquet: as, however, we do not perceive any thing very remarkable in it, we shall proceed to—

Chap. VIII.—*On the Ligature of Nerves.* Lesion of a soft part, produced by a ligature, is soon followed by infiltration, above, below, and around it, of an organizable substance, like that which agglutinates wounds: the tissues inclosed in a ligature inflame, soften, become liquified, so to speak, and are divided, more or less speedily, according to their nature and size; and the swelling and hardness of the surrounding parts gradually subside on the ligature being expelled, and go into that state which succeeds the healing of a wound made by a cutting instrument. The ligature of a nerve, however, presents some peculiarities: at the instant of its being tied, the nerve contracts distinctly; without doubt, says our author, from the interruption of continuity produced immediately in the softer parts of the organ: the membranous structure which remains inclosed in the ligature offers so much resistance that it is absolutely impossible to cut it through immediately, however great a degree of constriction be applied. Ligature of a nerve occasions intense pain, usually accompanied, in the lower animals, with excretion of urine and fæcal matter, and an interruption of the nerve's functions; when it is tied suddenly, the results are little different from those which proceed from division of the organ with a sharp instrument: several facts are adduced in support of these statements.

Valsalva and Morgagni tied the par vagum of a dog, and soon afterwards untied it, but the animal died in five hours: the nerve had become thicker than it was before the application of the ligature. The former ascertained, that dogs expire much sooner from ligature of nerves than their section: he tied the eighth pair, in a young bitch, near the larynx; the animal instantly lost her voice, and, although the ligature was soon removed, and the edges of the wound kept in apposition by Nature, she never recovered it: tormented with efforts at vomiting, this animal died in eight hours after the operation. Having tied the same nerves in a dog, the ligature, which he drew very tight, was instantly unloosed; nevertheless, the animal began soon to vomit, and expired in 14 hours, making strong attempts to evacuate the stomach, and discharging frothy blood from its mouth.

On removing a ligature, we observe a depression on the nerve, but at the end of a few days this is effaced, and the part appears thickened. Galen had remarked, that a tight-drawn ligature induces a partial section of the nerve; so that, though it be soon withdrawn, the functions of such nerve are not the less suspended: it is only in cases where the ligature has been imperfectly tied, or consisted of an elastic substance, that the nerve's functions have ever been regained: this fact explains the circumstance of Vesalius, Columbus, Casserius, and Rialamus, having known the voice to be restored, on untying a ligature from the recurrent nerve: it also explains the results of Bidloo's and Stokhausen's experiments, that ligature or even section of the sciatic nerve does not destroy motion and sensation in the *whole* limb. Ligature of a nerve, even a large one, our author says, does not give rise to the convulsions, spasms, and other "grave accidents," which pathologists have been in the habit of ascribing to it: when allowed to remain on the nerve, it has the same effects as an incision; it occasions a breach of continuity and suspension of function: this is exemplified in three "observations" from Mollinelli's work on aneurism, but we have not space for their transcription.

Dr. D. proceeds to observe, that a ligature applied to a nerve determines, at the same time, above, below, and around the place, an effusion of coagulable matter, and especially an "ovoid" enlargement in the *superior* end. There is error, therefore, he adds, in the statement of Bidloo and others, that this enlargement ("*gonflement*") exists principally *below* the ligature: this enlargement is quite manifest at the end of a few days, after which it becomes vascular: the ligature, at the end of an uncertain, but not long period, divides the nerve, and is itself detached: then the two ends, retained in exact contact, and, as it were, united externally by a thickening of the surrounding cellular texture, and the deposition of coagulable matter, soon re-unite in the centre of the separation from which the ligature had just been evolved. In such cases, there is never, as in section of the nerve, a separation or displacement of the two extremities: these remain always in perfect relation: for, when they have been totally divided by the ligature, they are already in part re-united around it. The swelling of the surrounding parts is gradually and completely resolved; that of the nerve, on the contrary, continues, principally above where the ligature was applied: this enlargement, says Dr. D. was found in the median nerve at the end of thirty years, after it had been tied along with the brachial artery: he views these facts as being established by the results of certain experiments and dissections, which afford some really interesting details.

When the ligature on a nerve remains in its place, the fact enters into the class of "Wounds of nerves complicated with the presence of Foreign Substances," and should be held as the cause of unfavourable symptoms. In some instances, a second amputation has been practised with the design of removing the violent pain experienced in the cicatrix of a stump. Sœmmerring ascribes pain of this kind to the tubercle which forms on the extremities of nerves divided in amputation; but it appears, from the researches of Van Horn and Breschet, that the tubercle is not the cause of such pain. When the ligature has been evolved, ought we not, our author enquires, to attribute the continuation of the pain to the existence of a fibrous cord comprised in the enlargement of the nerve in the tissue of the cicatrix? There can be no reason, he adds, why this phenomenon should not have place after section of a nerve with a ligature, as well as after its section by a sharp instrument. He then enumerates some "facts," to prove that, in proportion with the re-union of the ends of a nerve divided by ligature, its functions, at first suspended, are gradually recovered, and, in the issue, become perfect: one pathological history illustrative of this, from the practice of M. Richerand, deserves every consideration, but its length precludes our offering the homage of a transcription to its merits.

Some persons believe that the favourable results of a ligature on a nerve can only be obtained when, in the same parts, there are other nerves, which, by their anastomoses with the former, are enabled to supply their functions. In our author's mind, however, this "hypothetical proposition" is inexact; and he regards, as being more conformable to facts, the doctrine, that ligature, as well as every interruption of continuity and function in a nerve, does not endanger life, so long as such nerve is merely the "conductor" of sensation and motion; and that if the patient survives, the re-establishment of its continuity and functions takes place: nevertheless, he adds, some authors suppose that certain ligatures become more dangerous when anastomoses are defective.

The ligature, and every other division of a nerve whose functions are essentially vital, as the pneumo-gastric, cannot be made on both sides of the body at the same time without occasioning sudden death: the process of re-union is then impracticable. But if the experiment is performed on one side only, and the animal survives, re-union takes place, and in a reasonable time the same thing may be done on the other side.

Others have taught, that the simultaneous ligature of the principal nerve and blood-vessels of a member must necessarily be succeeded by its becoming gangrenous: but, says Dr. D. we should carefully ascertain how much truth there is in this general proposition, and how many circumstances, in such a complicated lesion, ought to be referred to each of the particular lesions of which it is composed. On this head, he comes to the three following conclusions: 1st. We know positively, that in a healthy subject, and not very old, the ligature of the chief artery of a member produces only a very "momentary" suspension of the circulation, which is soon re-established by the collateral branches: 2dly, It is absolutely the same in regard to a principal vein, although some modern surgeons have asserted the contrary: and, 3dly, It is likewise the very same with respect to an important nerve; its functions are recovered "some time" after it had been tied. Now, he inquires, what shall be the result of a simultaneous lesion of the vein, artery, and nerve? This question leads him to review the experiments instituted by Valsalva for the purpose of refuting Varignon's assertion, that animals die instantaneously of apoplexy, on both nerves of the eighth pair being tied: he holds the results to be quite conclusive, and proceeds to state the effects of three experiments practised by himself on living animals. In the first, the injuries were too complicated to lead to any positive result; nevertheless, although the dog survived for six days, gangrene did not supervene in the members whose nerves and blood-vessels had been tied or cut. In the second, where all the nerves of the brachial plexus, as well as the arterial and venous trunks of the same member, were altogether tied, palsy of the member ensued; but, fourteen days after the application of the ligature, no symptom of gangrene or of infiltration had appeared: the limb, however, remained in a state of flexion, and the paralysis continued. In the third, which consisted of the same operation as the last, a most acute pain was felt instantly on the ligature being applied, and palsy, also, of the member immediately supervened; but, by the fourteenth day, when the animal was killed, no troublesome symptoms had arisen. On inspection, the brachial plexus of nerves and deep-seated blood-vessels were still firmly enclosed in the ligature, but the member, although carefully examined, did not exhibit any morbid alteration whatever. In three other cases, Dr. D. isolated the sciatic nerve, and applied a ligature to its trunk; but in one of them only was the process followed by mortification of the extremity of the member: in this, the dog was old, and the nerve tied at its issue from the ischiatic notch. The chapter closes with the sketch of a case which Mr. Swan adduces, to shew how little capable the anastomosing branches are of transmitting the nervous influence after the division of a nerve with a sharp instrument, or a ligature which produces the same results.

CHAP. IX.—*Cauterization of Nerves.*—Cauterization, whether it results from the application of concentrated caloric, or of any caustic substance, produces an eschar, the separation of which is equivalent to a wound with loss of substance. Nerves are rarely, if ever, the exclusive subjects of burning or cauterization: when injured in this way by themselves, or simultaneously with other soft parts, they are converted into an eschar, on the detachment of which there remains, between their two ends, a space proportionate to the extent of structure thus destroyed; and, in general, the cicatrix that closes the wound does not restore the nerve's continuity or functions. For this reason cauterization is usually preferred in cases of neuralgy, and most frequently determines favourable results, while simple incision is too often followed by a relapse. Dr. D. induced this kind of lesion in animals; in one instance, where the cauterization was very superficial, the nerve's functions were not destroyed; in another, complete cauterization instantly occasioned palsy of the limb. The history of both these experiments is given, with an account of the nerve's appearances in the latter, on the twelfth day after it had been injured. The animal walked on three feet, the palsy of the other being complete; the nervous trunk was completely divided across, and a space of more than an inch existed between the two ends; the upper was slightly thickened and flattened; the lower exhibited no traces of having been cauterized; and both of them adhered strongly to the surrounding parts, with which they were intimately blended. Additionally to these, illustrative of the same doctrines, our author relates a case of maxillo-dental neuralgy cured by the actual cautery; the patient, a lady of 50, afterwards enjoyed the "best" health.

Chap. X.—*Re-union of Divided Nerves.*—When a nerve has, by any means, been perfectly divided, the two ends readily unite, if they are allowed to remain in apposition. This fact, which is common to all organized and living structure, is incontestable; but, says Dr. D. it has been the subject of much investigation to ascertain whether the cicatrix is of the same texture, and performs the same functions, as the nerve itself; and especially when there is distinct loss of substance, whether a regeneration of true nervous tissue really unites the two ends. Our author goes very comprehensively into this inquiry; and, for the sake of precision, divides this section of his book into two parts. In the *first*, he states the facts, opinions, doubts, and conjectures of the greater part of physiologists—including Fontana, Monro, Michaelis, Arnemann, Cruickshanks, Haighton, Reil, Meyer, Zimmermann, Sœmmerring, Meckel, Boyer, Richerand, Delpech, Callisen, Monteggia, Swan, and Breschet, who have written expressly or generally on the re-union and regeneration of nerves. And, in the *second*, he "exposes the results of his own observations." We must, however, pass over the former entirely, and restrict our account of the latter to a brief but faithful outline.

Re-union of the lips of a wound, our author observes, presents phenomena which are general or common to all the tissues and all the organs which have been simultaneously implicated. When a wound, for instance, divides the skin, cellular and subcutaneous adipose tissues, aponeuroses, muscles, nerves, blood-vessels, and lymphatics, and the edges of it are brought together and kept in exact contact, this wound, after the bleeding ceases, and the "traumatic pain" has subsided, if there was no accidental cause of irritation, agglutinates throughout by means of a viscid, colourless fluid, which is coagulable lymph, and consists of albumen and fibrin of the blood. This coagulable substance, this organizable matter, says Dr. D. constitutes the primary medium of all adhesions—of all re-unions; this agglutination soon gives place to a real vascular re-union; and this re-union, moreover, is every where identical; it appears to be formed of compact cellular tissue, interspersed with an immense quantity of small vessels. By and by, however, this re-union assumes new characters; between the edges of the skin it becomes a tissue analogous to the dermal;—between the ends of the muscles, a kind of fibrous "intersection;"—and between the extremities of a tendon, a genuine tendinous substance. Instead of the innumerable quantity of minute vessels which render it everywhere homogeneous, three or four principal "arteriolets" re-establish a communication between the two ends of the artery which had been divided: in fine, the same thing nearly has place with every organ and every tissue in particular, the rest of the cicatrix in general blending itself completely with that part of the cellular texture whose continuity it restores.

Divided nerves re-unite in the same way as all other parts, and their re-union is speedy and exact in proportion to the accuracy with which their ends are maintained in apposition. *Ligature* of a nerve is constantly followed by an exact re-union of the two ends, and by a very prompt recovery of its functions. *Imperfect section*, or *puncture* of a nerve, which has been accused, says Dr. D. of occasioning so many grievous accidents in man, does *not* occasion such accidents in the lower animals; its re-union, and the re-establishment of its functions, are speedy. *Complete section* of a nerve, in a part *not* subjected to much motion, is ordinarily followed very soon by an exact re-union, and the perfect recovery of its functions. *Complete section* of a nerve, in a part *subjected to much* motion, as in the vicinity of a joint, occasions, besides the primary, an accidental and variable separation; in such cases, the re-union, if it ever succeeds, is tedious and imperfect, and the restoration of function is also defective, or even never takes place. Finally, when there is *considerable destruction* of the nervous trunk, by excision, cauterization, or a contused wound, a great separation of its two ends remains, and its functions are for ever lost, whatever nerve be thus injured. This circumstance, says the author, proves that the nervous anastomosings do nothing towards the re-establishment of function. From all these facts, then, he adds, we may conclude, that nerves fairly divided do re-unite; and that, when re-union fails, this depends entirely on a considerable separation of the ends, induced by local motion, or by loss of substance.

When a nerve has been divided, an oozing of organizable matter takes place, for some days, around its ends, on its surface, and in the intervening space; the surrounding cellular tissue is penetrated by this matter, and loses its permeability; in this state, the ends of the nerve are simply agglutinated together and to the adjacent parts, and its functions are still suspended, as they were immediately after its division. Forthwith, the nerve's two ends, which are thickened, the investing cellular texture, and the organizable matter, acquire greater consistence, and become very vascular; in this state, which continues some time, the two ends of the nerve are united by an organized vascular substance, but still there is not yet a communication of nervous action between them. In due time the cellular tissue ceases to be compact and vascular; the intermediate substance gradually diminishes in size, consistence, and redness; acquires the appearance and texture of a nerve, and ultimately discharges its functions. This end is attained sooner and more perfectly, when the nerve has been simply divided, or the excised piece small, and in a part not much subjected to motion. On the other hand, when the separation has been important, re-union is never attained, or rather takes place through the cellular texture, which does not acquire, at a certain distance from each end, the nervous structure and qualities. The time necessary to a complete recovery of the structure and functions of a nerve has not been precisely ascertained. Dr. Descot imputes exaggeration to those writers who state its being several years; in his opinion, six or eight weeks are sufficient.

Division of the united pneumo-gastric and tri-splanchnic nerves, in the dog, uniformly occasion death, when it is practised simultaneously on both sides. It is on these nerves that the regeneration of tissue and the recovery of function can be most advantageously observed. Dr. Descot is convinced, by the results of many experiments, that it is neither by the interposition of a substance simply humid between the ends of a divided nerve, nor by the remote action of the nervous system, nor by the anastomosings of nerves, but by a real nervous cicatrix, that the re-establishment of nervous function is produced; it gradually and uniformly accompanies the progress of organic re-union. Five experimental histories, confirmatory of the preceding doctrines, complete this tenth division of the work. They exhibit the effects of nervous injury inflicted in various ways on living animals, and comprehend very minute but important details of the pathological symptoms and the organic lesions unfolded by dissection after death. We regard them as favourable testimonies of the author's zeal and science, and should willingly, but for their length, have inserted one or more of them in this article, in the form of a concise translation.

Chap. XI.—*Neuritis, Inflammation of Nerves.*—Nervous inflammation has been very seldom observed, and is generally confounded with the symptoms to which it gives rise. This, no doubt, depends on the difficulty of detecting its traces in the nerves, by reason of their whiteness, and the smallness of their blood-vessels: nevertheless, some observations in pathological anatomy do shew that they have really been inflamed. Reil states, that a man who died of typhus, after enduring exquisite pain in the nerves, had these organs deeply coloured with blood, and that, on the neurilema being destroyed with nitric acid, the pulp of the nerve was yellow, the blood having penetrated the medullary substance itself, imparted to this its colour. Our author has seen inflammation of nerves: constantly, he says, in the vicinity of a wounded nerve, the organ may be perceived, in a few hours, to be swollen, dark red, and very vascular: on such occasions, the nerve's functions become deranged or suspended, but are recovered as the traumatic inflammation subsides. Anatomical alterations are sometimes found in nerves, which should undoubtedly be regarded as signs of an antecedent inflammation more or less manifest; these may be serous infiltration of their cellular sheath—adhesion to the neighbouring parts—abscesses in the course of a painful nerve; softening and purulent disorganization—enlargement in the vicinity of diseased joints—circumscribed tumours—cartilaginous, osseous and calcareous transformations—and other morbid changes of structure. Inflamed nerves have been observed, on dissection, to be tumefied, reddened, and infiltered with a gelatinous fluid, their vessels being injected, and exhibiting a kind of varicose dilatation. In the femoro-popliteal nerve this injection was found exceedingly distinct, forming round, oval, very regular patches confined to the neurilema, while the nervous pulp itself was of a dull grey, and inelastic. In another instance, there was discovered on the same nerve, a nervous substance, shaped like a bead, the particles of which were hard, shining, fibro-cellular, and separated from each other by a soft deliquescent pulp, of a grey colour, inclining to red; the neurilema was thick, red internally, white and opaque on the outside. Finally, nerves have been seen enlarged, reddened, softened, with parts of them in the form of soft pultaceous swellings, from which oozed a "sanguinolent serosity," while their neurilema was granular, lamellated, opaque, injected.

As to the symptoms of neuritic inflammation, Dr. D. thinks they have been confounded under the name of pains, cramps, convulsions, emaciation, insensibility, palsy, neuralgy: nevertheless, he adds, it may be said in general, that the pain of acute neuritis is accompanied with a fever, which "is at least local." When this inflammation becomes chronic, the symptoms are altogether indistinguishable from those of neuralgy, "which is, perhaps, nothing else than a chronic neuritis," dependent on a particular organic lesion. Acute idiopathic inflammation of nerves is of very rare occurrence, but they are sometimes affected with the chronic kind, and this generally takes place at their ends in the stump of an amputated limb: in such cases, their size is increased to a certain distance, and their extremities assume the appearance of a gangliform tumour, very like the enlargement which forms the connecting medium between the two ends of a nerve which had been cut through. If we divide the nerve and tumour perpendicularly, we shall be able to trace the nervous fibres downwards into the tumour, and the coagulable lymph shall appear to be deposited between them: but, if the section is horizontal, the tumour presents a compact and regular aspect, like a piece of cartilage. In many cases of the ischiatic malady, Dr. D. believes the sciatic nerve to be the seat of the disease: the pain, in general, follows the course of the nerve so exactly, and the adjacent parts are so entirely free from every pathological appearance, that he must regard the nerve alone as the seat of the pain; and the affection itself seems to him to arise from an inflammatory state of the neurilema, which often terminates in the effusion of a serous fluid. Nothing is proposed as a mode of treatment, we therefore follow him to—

Chap. XII.—*On Ulceration of Nerves.* Seven pages of the eight wherein this section consists, are occupied with extracts and cases from Mr. Swan's book, with which our readers are already acquainted: we need not, therefore, advert to these, but may notice the observations of two "eminent authorities," with the author's concluding remark. Bichat states, that alteration never takes place in the nervous tissue, nor is this the seat of tumours, funguses, ulcerations, as it does in the systems whose organic properties are predominant: on the other hand, Béclard, in his addition to Bichat, declares, that suppuration, gangrene, ulceration, may occur in nerves, but they are never so affected primarily. With this last opinion our author is agreed: he concludes, that ulceration of a nerve is a very rare, but at no time a primitive affection; and mentions the circumstance of Béclard's having often said to him, that he regarded "isolated" (idiopathic) inflammation of a nerve as a thing whose existence is exceedingly problematical.

Chap. XIII.—*Tumours of Nerves.* "Neuromes," or tumours of nerves, very improperly denominated ganglions, from the alterations of tissue which most frequently occur in these organs: Dr. Descot distinguishes them into two kinds, having relation to their size and situation, each of which he describes in a separate section.

§ 1. *Painful Subcutaneous Tubercle.* These are roundish and a little flattened; their size varies from that of a millet-seed to a bean; they are situated in the sub-cutaneous cellular tissue, on all parts of the body, but most frequently on the extremities; they are generally solitary, but in a few rare instances two or three of them have constituted a group; usually they are inclosed in cellular membrane, and adhere to the neighbouring parts by nervous filaments; on other occasions, they are situated in the substance of the nerve itself, whose fibres divide and surround them; and sometimes they have a very intimate connexion with the nearest vein. In a great number of cases, they appear in the form of a small fibrous cyst, inclosing a loose concretion, while the surrounding cellular texture and the skin retain their natural state. Commonly they are whitish, occasionally brownish, internally or at their surface: their consistence is usually firm, sometimes cartilaginous: in general, they are so small as to be perceptible only to the touch they occur much less frequently in men than in females, in early than in advanced life: their causes are various and uncertain: their formation and growth are very rapid, till they have attained a certain size, when they remain almost stationary for many years.

Unimportant as these small tumours seem, they often occasion intensely acute pain, of which the paroxysms have uncertain returns: this pain is exquisite in the tumour which is its seat, and darts instantaneously

(*"like an electric commotion"*) into the neighbouring parts, especially in the course of the nerve: it is slight at the beginning of an accession, but gradually becomes excruciating: its returns vary from ten minutes to two hours; but the frequency and intensity of these increase proportionately to the duration of the disease: some patients often sustain such seizures during sleep, from which they awaken with a start. Ordinarily, these tumours cause little inconvenience; but, during an attack, they, and the skin over them as well, are distressingly sensitive, and this sensibility is sometimes materially increased by the atmospheric changes: then, also, in certain persons, they have been observed to increase in size, and to exhibit a purple or blueish speck: in a few instances, they spontaneously disappear; but, unless they be extirpated, they most frequently accompany the sufferer through life. The remedy is excision and treatment of the wound for a cure by the first intention. Twenty-one cases, from Portal, Cheselden, Camper, Siebold, Newmann, Wood, Thompson, Newbigging, Gillespie, Bisset, Pearson, Hall, Windsor, Nicod, Marjolin, form the illustrative details of these doctrines; but to them all we cannot do more than refer.

§ 2. *On Voluminous or Clustered Tumours of Nerves.* Tumours of this kind are usually formed of a scirrhous tissue, varying in firmness, and interspersed with vesicles or small cysts, inclosing a syrupy fluid: sometimes, each of them consists of a single cavity, whose sides are fibrous or cartilaginous, and its contents a thin fluid; at other times, it appears to be of the "encephaloid tissue," with circumvolutions and vermicular elevations: oftenest, the nerve can be traced to the surface of the tumour, to which it furnishes an envelope. Most frequently, these tumours are moveable *laterally:* but when drawn *longitudinally,* in the course of the nerves, they cause much pain; the skin over them is generally healthy: they almost all appear to have an origin and cause which are unknown or very obscure: they have not been observed before the age of puberty, and are met with in men much more frequently than in females. Unlike the subcutaneous tubercles, they usually increase very rapidly in size, and undergo successive augmentations or "degenerations" of their structure: they are, for the most part, very painful. When susceptible of cure, extirpation is the means to be employed.

Dr. Descot has exercised much industry in arranging the comprehensive enumeration of anatomical and chirurgical facts, which, in the next part of his 13th chapter, constitute the foundation of his sentiments, now before our readers, regarding the nature and seat of the large-sized or clustered variety of nervous tumour: we refer to them, with approbation, as sketches of much value in the history of pathology: they are followed by the details of five select cases, descriptive of the characters and treatment of the same important lesions.

CHAP. XIV.—*Neuralgy.* We confine ourselves, at present, to Dr. Descot's observations on the neuralgic disease. It is, he says, a painful affection of a nerve, distinguishable by the seat of the pain, which is always fixed over a nerve, and by its nature extremely acute and lancinating. In common with other lesions of nerves, it may affect the "nervous cords," the plexus and ganglions, and the nerves which terminate in particular organs. Affections of determinate nerves, which form the subject of this chapter, are alone distinct; the rest are generally confounded with loss of function, or an organic change: and, indeed, if those of the nerves of the face and extremities, especially the lower, be excepted, all others are exceedingly obscure, uncertain, and difficult to be distinguished.

The subcutaneous nerves, particularly those of the face, are most liable to this affection: their superficial situation exposes them much to mechanical lesions or impressions of the atmosphere. Neuralgy most commonly results from an exposure to cold, or the suppression of a sanguineous, serous, mucous, or purulent discharge: it often exists in persons who are subject to other "moveable" diseases, as rheumatism or gout. Women, particularly after their critical period has past, are less frequently affected with it than men: it is sometimes caused by the presence or contact of an extraneous substance, or another diseased organ: in many instances, it proceeds from mechanical lesion; often from a small cartilaginous tumour, or a cancerous degeneration, compressing or affecting a nerve. Occasionally, says Dr. D. the cellular sheath of the lesioned nerve has been found injected with a serous infiltration, or even with suppurative inflammation, and the nerve itself having a reddish colour. In fine, he adds, in all other cases, the very vascular texture of nerves, the nature of the causes which determine neuralgy, the phenomena that accompany the paroxysms of pain, all conspire in suggesting the idea that the disease is nothing more, at least in many instances, than an intermittent or remittent inflammation of a nerve. Dr. D. treats of five kinds of neuralgy, the chief of which we shall briefly notice.

*Facial Neuralgy.* This form of the disease, our author observes, presents accessions of pain, with intervals of ease, and the seizure ordinarily begins with the violence of an electric shock: it is, however, announced sometimes by the "perception of an odour," an itchiness in the disease's seat, a kind of *aura epileptica*, a palpitation or "formication" in the eyelids, a particular sense of tension in the palate and nose, or by a distressing contraction of the arm: sometimes, the pain rests in a circumscribed point; at other times, it is widely diffused: it occupies, in some instances, the orbito-frontal branch of the trifacial nerve; in others, the submaxillary branch; and, in others, the inferior maxillary; but it may affect the whole of one of these three nerves, and all their ramifications. Most frequently, it is confined to their superficial or facial portion: occasionally, it occupies but one branch, or even a single fibre of a nerve only: in other cases, the whole of one side of the face, most generally the right, very rarely both sides, are affected. Ordinarily, the pain is fixed: sometimes variable, and, as it were, fluctuating between different parts: it has been known to leave one side of the face and attack the other. Some writers, says Dr. D. have said that it affects the facial nerve; but they have not related any distinct examples of it: be that as it may, he adds, "its situation can scarcely be compared to any other." It surpasses, "in atrocity," the most violent pains of tooth-ache or ear-ache: the patient is in "real despair;" ordinarily, he can "neither speak, nor chew, nor swallow:" some persons, however, have the lips and tongue constantly agitated by particular motions, accompanied with noise, and resembling that which is caused by "manducation;" these can both chew and speak.

Strong pressure, in general, can be better endured on the affected parts, in this form of the disease, than a slight touch: the muscles of the face, especially those of the lips, are often disturbed by convulsive motions or spasmodic contractions: occasionally the patients fall to the ground, or on their knees, and are seized with general convulsions. Most frequently, during a paroxysm, the countenance is red and swollen; sometimes it is pale, and grows livid: in other cases, the blood-vessels of the affected part are distended: our author has seen the temporal artery, in particular, become very large, and be stretched like a cord: red streaks, also, are "sketched" on the skin of the forehead, the nose, and the gums: there is little fever, and the pulse is regular, often slower than in health: in general, the seizure is short in proportion as the pain is exquisite: commonly it lasts for a few minutes only, seldom for fifteen minutes, very rarely for a whole hour; it subsides with a gradual, sometimes a sudden decrease of the pain, followed, occasionally, with a copious flow of saliva, tears, mucus from the nostrils, and eructations. The intervals between the paroxysms are often "regularly equal," often prolonged, without any regular type, for hours, days, weeks, months, or even years. Women appear to be more frequently affected with "*prosopalgy*" (Dr. D.'s new name for neuralgy) than men: it occurs sooner in the former than in the latter, in the decline of life: affects the rich in more instances than the poor: is seldom met with in hospitals: prevails most in cold countries; and, while it may sometimes attack persons enjoying perfect health, more frequently seizes those who are affected with piles, gout, diseases of the abdominal viscera, derangement of periodical discharges, and cancerous maladies. Among its chief exciting causes may be ranked, injuries of the head; suppression of evacuations, natural, artificial, or morbid; effects of exposure to cold; and depressing affections of the mind. When the disease already exists, accessions of it are often induced by anger, terror, a bright light, using frictions on different parts of the body, or touching them, especially those that are affected by manducation, speaking, and laughter. Pathologists have essayed to discover its proximate cause in the arthritic, catarrhal, and carcinomatous "vices," in a topical affection of a nerve, or an "alteration of the osseous canal" through which it passes, and, finally, from a direct asthenia; from all which an "augmentation of *incitability*" should result. The disease consists in paroxysms, and is not necessarily accompanied with fever ("*apyrectique*"); it is difficult, says Dr. D. to rank it constantly among the inflammatory affections. Prosopalgy in part, he adds, exhibits many varieties, according to its causes; 1st. It appears sometimes to be traumatic; 2d. Inflammatory, in those cases wherein it succeeds a sanguineous evacuation, and is accompanied by a "*local fever*;" 3d. It seizes persons subject to rheumatism, and then it assumes the "moveable" character of that disease; 4th. In some instances it is "*metastique*;" 5th. It is occasionally connected with abdominal affections; 6th. At other times it attacks the gouty; 7th. It is sometimes an accompaniment of carcinomatous diseases; 8th, and of the syphilitic; 9th. Frequently, in fine, it depends, like the other neuroses, on an irritation of the nerves, the *material* cause of which is very obscure. It is exceedingly unsusceptible of a cure; nevertheless, adds the Doctor, there are examples of its having been cured; but, as it is periodical, we should be careful not to mistake an interval for a complete cure. Its cure is sometimes accompanied with a cutaneous eruption, or a critical discharge: occasionally, persons cured of it become affected with abdominal dropsy, cancer, or some other "cerebral neurosis." Dr. Descot laconically concludes—"for the cure of this affection, they have employed a crowd of dietetic, pharmaceutical, and chirurgical remedies." Next follow one "observation" of the *facial*, one of the *maxillary*, and three of the *sub-orbitary* forms of the neuralgy; and this brings us to that of the "*members*," of which we shall give a sketch.

*Neuralgy of the Extremities.* This occurs oftenest in the ischiatic or femoro-popliteal nerve; sometimes in the crural, in the cubital and radial, and in the soles of the feet: it has also, now and then, been observed in some of the nervous branches of the extremities. In *ischiatic* neuralgy, the pain arises between the great trochanter and the ischium, shoots upwards to the sacrum and the loins, and particularly downwards, to the posterior and superficial part of the thigh, as far as the hollow of the ham: but it does not always confine itself to these parts, says our author, for it may extend from the external tuberosity of the tibia to the malleolus externus, following the external edge of the crest of that bone, and terminating in the sole of the foot; the femoral muscles become flabby, and the whole of the limb grows lean. *Crural* neuralgy is recognized by a pain which begins in the groin, descends along the internal face and surface of the thigh and leg, and ends in the back of the foot: sometimes it originates in the toes, and ascends to the inguinal region. In all cases of this form of the disease, the pain undergoes exacerbation at night; and, in the ischiatic kind, it returns periodically, especially so long as the affection is recent. The pain he suffers is so violent, that the patient cannot remain in bed, the heat of which is intolerable: neither can he sustain himself on the affected limb, though it still preserves the power of motion unimpaired: this, however is seized with cramps and tremblings; and, if the sick person attempts to stand on the sole of his foot, it becomes livid; and the veins, extensively distended, acquire a great resemblance to metallic cords. Fever does not accompany the disease in its first stage: in time, the pain decreases, then grows more obtuse, and finally disappears; but then, the limb *first* exhibits flaccidity of the gastrocnemial muscles, and, *ultimately*, progressive emaciation. The general health is "scarcely affected" by the crural and ischiatic neuralgies; nevertheless, people having violent attacks, experience inappetency, epigastric pains, attended with squeamishness. The disease may prove the cause of palsy, and atrophy of the lesioned parts. In *cubital* neuralgy, the pain begins in the arm-pit, and, most commonly, reaches to the bend of the arm, sometimes even to the hand, which it renders œdematous: generally, it is most severe in the space between the arm-pit and the elbow, particularly on its being touched: it is ordinarily periodical: in all cases, the exacerbations take place during the night, and occasion sleeplessness; the patient raises his arm, during a paroxysm, compresses the swollen hand with the other and complains of a sense of anxiety almost indescribable: accidental emotions readily induce paroxysms, or render the existing one more exquisite: when the disease is recent, the temperature of the arm evidently rises; if confirmed, the arm shrivels, and the skin on the inside of the hand becomes wrinkled. The cubital and the ischiatic nerves appear to be more subject to neuralgy than any others; because, throughout a very long course, they are covered only by a small number of muscles, and, in consequence, are exposed to external lesions: moreover, the neurilema of the sciatic nerve is so loose as to leave sufficient space, between itself and the pulp of the nerve, for the admission of morbid depositions; besides, it receives arteries which "facilitate in it the development of inflammation." Ischiatic neuralgy is more common in man than in females: it assails both sides indiscriminately, but rarely occupies both extremities at the same time. The exciting causes of neuralgy of the extremities, are, wounds and contusions; exposure to cold; lying on the cold ground, especially when the body is heated; a morbid metastasis; suppression of the hæmorrhoidal and lochial discharges, or of the secretion of milk; and particularly gout. The crural may proceed from puncture of the saphenal nerve in venesection, and, also, sometimes from an affection of the uterus. Two practical "observations" explain this description, but these we cannot copy.

*Neuralgy of the Trunk.* Several cases of this affection in nerves of the sides of the chest and abdomen have been described by pathologists. A young woman, on the subsidence of her monthly discharge, was seized with an acute pain between the eighth and ninth rib: it followed the distribution of the intercostal nerve, returned in irregular paroxysms, and continued till her death. On dissection, the nerve was found to be reddened and shrunk. For several years past, Professor Fouquier has distinguished neuralgies of the chest; and M. Nicod, has published some cases, and, at the same time, stated that he has seen *more than two hundred.* He says that they occur fifteen times in the left side for one in the right—that they are very rare in men—have almost always their seat near the anterior extremity of the seventh, eighth, and ninth ribs—and that they are usually complicated with similar pain in the mamma, the epigastrium, the bowels, or the uterine dependencies. He believes that this affection has often been misunderstood, and that many practitioners have regarded it as an *abdominal inflammation.* Professors Chaussier, Richerand, and Delpech, have observed an ilio-scrotal neuralgy: its seat is in the anterior branch of the first lumbar nerve; it stretches along the haunch, hip, and groin, and terminates in the scrotum or labia of the vulva; it differs from nephritis in there being no alteration in the urinary secretion. M. Coussays has described, under the name of Lumbar Neuralgy, a pain which had its seat in the haunch, and in the posterior branch of the first lumbar nerve. The pain underwent violent accessions, was always attended with vomiting, and sometimes followed by diarrhœa, head-ache, and fever: the accessions lasted several days. It appears to me, says Dr. D. that this affection was an "ureteritis," and not a neuralgia.

CHAP. XV.—*Local Paralysis.*—Paralysis, taken in its general acceptation, is the "abolition" of motion or sensation, or of sensation and motion both together: it may be universal, and then it almost immediately terminates in death: when it affects the "muscles of the skeleton," it constitutes *external* palsy: it is *internal* when seated in the viscera: it may attack one side of the body only, the inferior portion of the trunk and the abdominal extremities, or one of the arms and the leg of the opposite side. Cases of this kind, of course, do not enter into the subject of this chapter, inasmuch, says the author, as they depend on an affection of the spinal marrow or the brain.

Local palsy most frequently affects the muscles of the face—sometimes those in other parts of the body; it depends generally on a local affection of the nerves. Many instances of the facial kind are related in books—Dr. D. has seen a "certain" number. When one side of the face is affected, as it often is, and the right especially, the disease constitutes a facial hemiplegy. Usually it comes on suddenly; in other cases it is preceded by a kind of acute rheumatism of the face, or rather a rheumatic facial neuralgy: the face is then drawn towards the affected side, and often expresses the sardonic grin. Persons suffering from this affection, lose the power of raising or depressing the eyebrows—of closing or opening the eye-lids—of elevating the nose or lip—of dilating the nostrils—of raising the angle of the lips—of shutting the mouth—of retaining the saliva or food—or of turning under the teeth the portions of food which accumulate between them and the diseased cheek. Mastication is seldom impeded, and the tongue remains unaffected, except in seizures of apoplectic hemiplegy. When the disease continued through life, different lesions of the facial nerve have been detected, and also of the parts which surround it, as, thickening of the sheath of the nerve, necrosis of the "spiroid canal" of the temporal bone. In other cases, a portion of the nerve has been cut out, in extirpating a deep-seated tumour in the parotid region. The weight of such a tumour, by compressing the nerve, has induced a facial palsy. Very generally the disease arises from sudden exposure to cold, and then it appears to depend on a neuritis; it commonly subsides with equal rapidity. In persons having rheumatism in other parts of the body, it sometimes supervenes without any manifest cause. Suppression of an exanthematous eruption, and, indeed, all the causes of inflammation, may be the causes of this affection. It has more than once been the result of a severe blow on the head.

Local palsy, moreover, occurs in the muscles of the head and neck, in those of the thorax and abdomen, and in those of the extremities, the upper as well as lower. It has been observed as a sequela of "saturnine colic" (*col. pictonum*), of dysentery and "other" affections of the colon, and of diseases occasioned by worms; in all which cases it appears to be transmitted through the abdominal plexus, especially the intestinal, to other nerves, by means of their anastomotic communications.

Local affections of nerves may give rise to a multitude of neuroses, and other general affections. *There is not, indeed,* says Dr. D. *one nervous affection, generally considered, that may not be determined by a local affection of a nerve.* Thus, besides the neuralgy and palsy just described, convulsions, epilepsy, tetanus, and all the other neuroses, are often occasioned by the mechanical lesion, inflammation, the transformation of its texture, or, indeed, the "degeneration" of a nerve, or from some analogous affection of the parts immediately adjoining. Nevertheless, as these neuroses often depend also on perceptible alterations of the "nervous centres," they are usually regarded as general affections of the nervous system; for which reason, says the author, we shall dispense with speaking of them in this dissertation. Dr. D. next proceeds to give what he calls a very curious case of alteration in the third pair (*trijeaumeau*) of nerves, from the practice of M. Serres, and then concludes his volume with some "observations" of local palsy. The first of these, and that communicated by M. Serres, are valuable pathological documents, and may be consulted with advantage in the original.

We regard this dissertation of Dr. Descot's as a commendable essay towards the advancement of pathological history, and, consequently, as being in some degree adapted to facilitate the study of this branch of medical science; for this reason, we have thought it right to exhibit a comprehensive view of his doctrines, leaving our readers to examine, generally, the facts which the author has selected for their foundation. In all that concerns the treatment of the local affections of nerves, the book is useless; but the Doctor has not undertaken to discuss this difficult question, and assuredly he had a right to choose his own subject. We close his book, in fine, with sentiments of gratitude and respect; and our desire is, that he may be inspired with a large portion of his patron's spirit.

